# Identification and Comprehensive Evaluation of Drought Tolerance in Sorghum During Germination and Seedling Stages

**DOI:** 10.3390/plants14121793

**Published:** 2025-06-11

**Authors:** Manhong Wang, Irshad Ahmad, Bin Qin, Lei Chen, Weicheng Bu, Guanglong Zhu, Guisheng Zhou

**Affiliations:** 1 Joint International Research Laboratory of Agriculture and Agri-Product Safety, The Ministry of Education of China, Yangzhou University, Yangzhou 225009, China; mx120230760@stu.yzu.edu.cn (M.W.); irshadgadoon737@yahoo.com (I.A.); 18306330657@163.com (B.Q.); clyzuu@163.com (L.C.); mz120231391@stu.yzu.edu.cn (W.B.); 2 Jiangsu Provincial Key Lab of Crop Genetics and Physiology, Yangzhou University, Yangzhou 225009, China; 3 College for Overseas Education, Yangzhou University, Yangzhou 225000, China

**Keywords:** sorghum, drought tolerance, variety screening, comprehensive evaluation

## Abstract

Drought is a major factor limiting crop growth and yield. Enhancing drought resistance is an important strategy to sustain higher yields, with an emphasis on developing drought-tolerant cultivars. In this study, 19 sorghum varieties from both domestic and international sources were selected as experimental materials. At the seedling stage, 11 above-ground and below-ground phenotypic traits were investigated. Under 25% PEG-6000 concentration, drought tolerance during the seedling stage was assessed using differential analysis, such as correlation analysis, principal component analysis (PCA), membership function analysis, regression analysis, and cluster analysis. The present results demonstrate that the principal component analysis could represent 77.18% of the data from the original 11 indicators. Total root length, stem diameter, and leaf area were identified as the main evaluation indicators for sorghum seedling drought tolerance. In addition, based on principal component scores (F) and drought tolerance metric values (D), the 19 sorghum varieties were classified into three categories through systematic cluster analysis: two varieties were classified as highly drought-tolerant, nine as moderately drought-tolerant, and eight as drought-sensitive. Two varieties, such as Longza 24 and Jinza 12, were identified as drought-tolerant during the seedling stage and can serve as valuable resources for evaluating drought tolerance throughout the full growth period and for breeding improvements. Hence, this study established a drought tolerance evaluation method by integrating above- and below-ground phenotypic traits indicators, providing theoretical support for the identification of drought-tolerant sorghum.

## 1. Introduction

Drought is a widespread abiotic stress that has significant impacts on agriculture, ecosystems, and food security [1]. Recent studies have shown that drought stresses at different developmental growth periods severely hinder crop physiology activities and, as a result, delay maturation and cause substantial yield losses [2,3]. Over the past two decades, the duration and frequency of global drought stress have increased by 29% [4], making Asia one of the most impacted regions [5]. Recently, drought stress has resulted in crop loss exceeding 30 million tons, making it one of the most severe threats to China’s food security [6]. Therefore, the screening of drought-tolerant varieties is crucial in agricultural production to avoid crop loss during abiotic stress.

Sorghum (*Sorghum bicolor* L.), a C4 crop with high energy, is characterized by a short growth cycle, high biomass yield, and stress resistance [7]. It is a valuable source of grain, forage, and sugar. Sorghum is an excellent drought-tolerant crop due to its diploid genome structure and efficient photosynthetic system [8]. Identifying drought-tolerant sorghum varieties is essential for maximizing land use in arid regions to ensure food security for future demands. Polyethylene glycol (PEG) has been widely used to simulate water stress [9,10]. The most evident effect on plants is the inhibition of growth under drought stress conditions [11]. Drought stress induced by PEG significantly declined both the speed and percentage of sorghum seed germination. Post germination, drought stress markedly inhibits the growth of roots and shoots [12]. Previous studies demonstrated that drought stress suppresses various aspects of plant growth, including plant height, fresh and dry weight, leaf number, leaf area, root length, and the number of root systems [13,14]. Therefore, using multiple morphological indicators to evaluate plant drought tolerance is a positive approach. For instance, the number of root systems, leaf number, and leaf area can be used as indicators for assessing drought tolerance in kiwifruit, as well as leaf length, plant height, and stem diameter in sugarcane [15,16]. These findings indicate that the selection of drought resistance indicators should be crop-specific.

Previous studies on screening drought-tolerant sorghum varieties have been relatively limited. Negarestani et al. [17] identified two drought-tolerant genotypes among five sorghum varieties using multivariate factor analysis and stress resistance scoring. Vijayalakshmi et al. [18] found that chlorophyll content could serve as an early screening trait for sorghum selection. However, these studies conducted drought tolerance screening throughout the entire growth period, resulting in excessively long evaluation cycles that hinder rapid identification of strongly drought-tolerant varieties. Moreover, the limited number of genotypes examined prevented comprehensive screening. The germination and seedling stages represent critical phases in crop growth, significantly determining population size and structure. Nevertheless, research evaluating drought resistance during these early growth stages in sorghum, particularly for screening different genotypes at the seedling stage, remains scarce. Establishing drought tolerance assessment during sorghum’s germination and seedling phases would undoubtedly facilitate the development of drought-resistant cultivars. For other crops, drought tolerance is typically evaluated by calculating comprehensive drought resistance indices incorporating individual morphological/physiological parameters and all measured traits [15,19,20]. Compared with physiological indicators, morphological traits are more readily observable and quantifiable. Most importantly, the improvement of morphological and agronomic traits consistently represents the core objective in crop breeding programs [21,22]. Therefore, employing morphological indicators to screen drought-tolerant sorghum varieties holds greater practical significance. In this study, we selected sorghum varieties with diverse genetic backgrounds to evaluate drought tolerance during germination and the seedling stages. Through measuring 11 morphological indicators, including germination potential, germination rate, seedling height, stem diameter, and above-ground fresh weight, we applied multiple analytical approaches (difference analysis, correlation analysis, principal component analysis, membership function analysis, regression analysis, and cluster analysis) to identify the most drought-resistant varieties. This systematic methodology provides a theoretical foundation for breeding drought-tolerant sorghum cultivars.

## 2. Results

### 2.1. Germination Potential and Germination Rate

**Figure 1:**
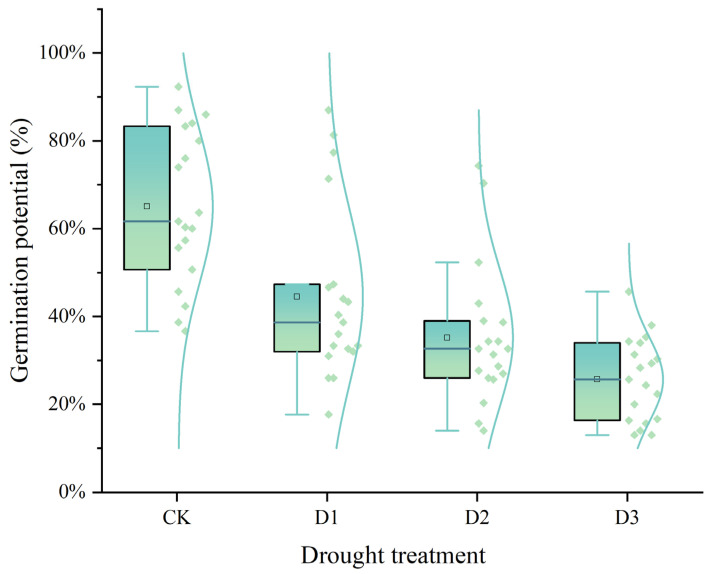
Effects of different CK, D1, D2, and D3 treatments at different concentrations of PEG-6000 0%, 15%, 20%, and 25% on the germination potential of sorghum seeds. The dots in the figure indicate the germination potential, and the lines indicate the distribution pattern of the 19 varieties under different drought stress.

The box plot demonstrated the effects of different drought stress treatments (CK, D1, D2, D3) on seed germination potential. As the PEG concentration increased, the germination potential of all 19 sorghum varieties showed a downward trend, with the most significant impact at the D3 concentration (Figure 1). Among them, Hunni showed the highest germination potential by 45.67% (a 50.53% reduction compared with the control), while LZ35 and LZ27 showed the lowest germination potential by 13.00% (with 84.40% and 74.34% reductions, respectively). At D2 concentration, LZ24 showed the highest germination potential by 74.33% (a 14.56% reduction), and LZ27 showed the lowest germination potential by 14.00% (a 72.37% reduction). At D1 concentration, LZ24 maintained the highest germination potential by 87.00% (unchanged from the control), and LZ27 had the lowest germination potential by 17.67% (a 65.13% reduction) (Table 1).

**Figure 2:**
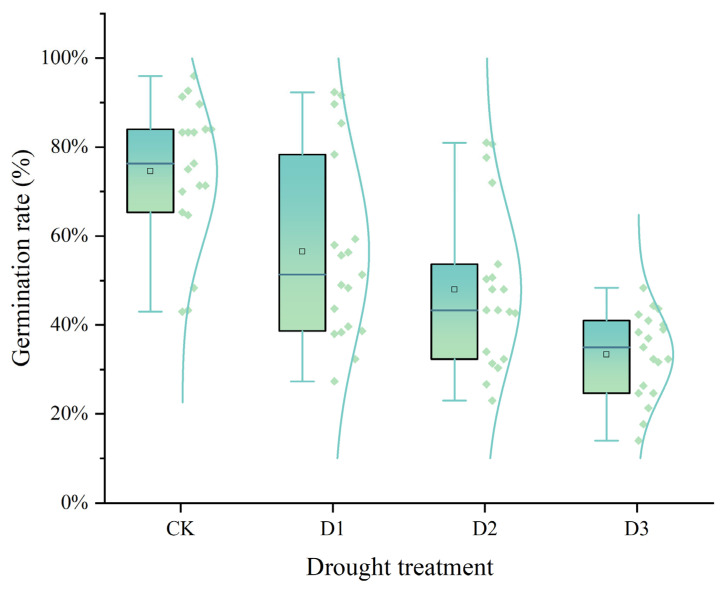
Effects of different CK, D1, D2, and D3 treatments at different concentrations of PEG-6000 0%, 15%, 20%, and 25% on the germination rate of sorghum seeds. The dots in the figure indicate the germination rate, and the lines indicate the distribution pattern of the 19 varieties under different drought stress.

Moreover, the germination rates of all varieties also decreased with increasing drought stress (Figure 2). However, at D1 concentration, LZ24 showed a higher germination rate as compared with the control. The most significant reduction in germination rates was observed at the D3 concentration. At this concentration, LZ27 had the smallest reduction at 14.00%, and Hunni had the highest germination rate at 48.33% (a 49.66% reduction). At the D2 concentration, LZ24 had the highest germination rate at 81.00% (an 11.33% reduction), and LZ27 had the lowest at 23.00% (a 64.43% reduction) (Table 1).

Table 1 compares the relative germination potential and relative germination rate of 19 sorghum varieties under three drought stress levels (D1, D2, and D3) and gives a composite ranking, with DXN ranking first, followed by JZ35 and LZ24. In D3 treatment, the relative germination potential of 13 varieties and the relative germination rate of 14 varieties were significantly reduced (*p* < 0.05). It indicated that drought severely inhibited sorghum germination.

### 2.2. Phenotypic Attributes

Under control conditions, 11 phenotypic attributes were measured and analyzed across 19 sorghum varieties. The coefficient of variation (CV) for these varieties ranged from 0.19 to 0.39, indicating that the selected sorghum varieties possess rich genetic diversity and are highly representative, thereby meeting the experimental requirements. Under drought stress conditions, the coefficient of variation for the measured attributes across different sorghum varieties ranged from 0.09 to 1.20, indicating that the selected indicators are sensitive to drought stress and exhibit significant variability among the varieties, fulfilling the experimental objectives. At D3 drought stress level, the reduction in phenotypic attributes in all indicators was most pronounced (Table 2).

### 2.3. Single Drought Tolerance Coefficient and Correlation Analysis

Single drought resistance coefficient (a) was calculated at D3 concentration, and the (a) value varied greatly among the 11 indicators for the same variety, which indicated that the individual indicators had different degrees of response to drought. For instance, JZ12 had the highest drought tolerance coefficient for plant height (0.50), while BK had the lowest (0.07). JHY2 had the highest coefficient for stem diameter (0.89), with BK at the lowest (0.27). LZ35 had the highest for leaf area (0.55), and BK the lowest (0.003). Ranking sorghum varieties by the comprehensive drought tolerance coefficient, JZ12 topped the list, JL6 ranked second, and BK was the lowest, with JA60 dying at this concentration (Table 3).

**Figure 3:**
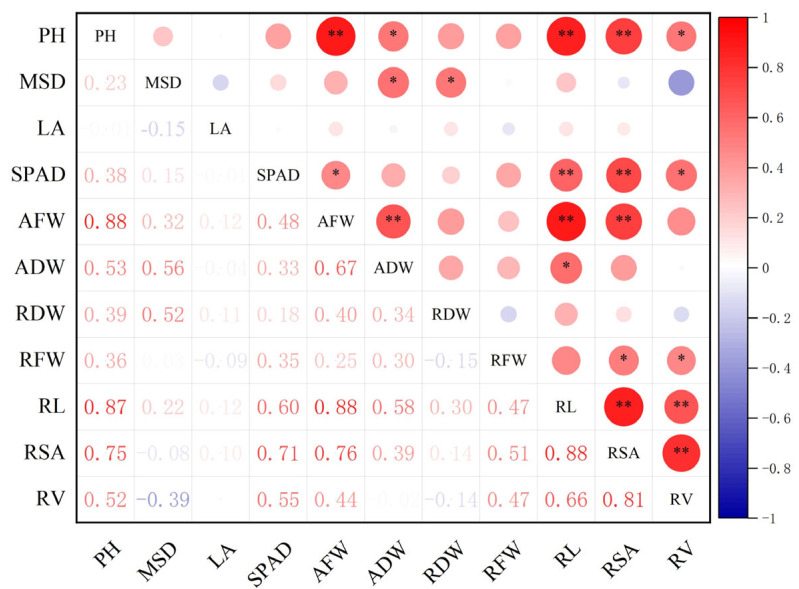
Single drought tolerance correlation analysis of sorghum varieties for each attribute. The notes * and ** in the figure indicate statistical differences at the 0.05 and 0.01 probability levels. PH, plant height; SD, stem diameter; LA, leaf area; AFW, above-ground fresh weight; ADW, above-ground dry weight; RFW, root fresh weight; RDW, below-ground dry weight; SPAD, relative chlorophyll content; RSA, total root surface area; RL, total root length; RV, total root volume.

A correlation analysis of the 11 drought tolerance traits was performed for a more accurate analysis. Most drought tolerance-related indicators had significant or highly significant positive correlations with others. Among the 19 varieties, plant height was positively correlated with above-ground fresh weight, total root length, and root surface area (*p* < 0.01), with correlation coefficients of 0.88, 0.87, and 0.75, respectively, and positively correlated with above-ground dry weight and root volume (*p* < 0.05). Similar positive correlations were found among other traits, such as SPAD value with total root length and root surface area (*p* < 0.01), and above-ground fresh weight with above-ground dry weight, total root length, and root surface area (*p* < 0.01) (Figure 3).

### 2.4. Principal Component Analysis of Drought Tolerance Attributes

Based on the PCA of 11 drought tolerance attributes across 19 sorghum varieties at the D3 level, the cumulative contribution rate of the first three factors (denoted as F1, F2, and F3) reached 77.18%. The eigenvalues of F1, F2, and F3 were all greater than 1, with values of 5.13, 2.18, and 1.18, respectively. The 11 original attributes were transformed into three relatively independent drought tolerance indicators. These three comprehensive indicators can serve as effective criteria for evaluating the drought tolerance of different sorghum varieties. Specifically, total root length and root surface area had high loadings in the F1 principal component; stem diameter had a high loading in the F2 principal component; and leaf area had a high loading in the F3 principal component (Table 4).

Based on the PCA scores, the calculation formulas for the principal components were derived as follows:

First principal component (F1):

F1 = 0.391X1 + 0.109X2 + 0.025X3 + 0.305X4 + 0.399X5 + 0.279X6 + 0.230X7 + 0.153X8 + 0.427X9 + 0.403X10 + 0.290X11

Second principal component (F2):

F2 = 0.067X1 + 0.568X2−0.029X3−0.101X4 + 0.14X5 + 0.353X6−0.221X7 + 0.457X8−0.015X9−0.222X10−0.459X11

Third principal component (F3):

F3 = 0.054X1−0.238X2 + 0.786X3−0.083X4 + 0.134X5−0.189X6−0.416X7 + 0.281X8 + 0.067X9 + 0.067X10 + 0.023X11

The comprehensive score (F) for each principal component was calculated as:

F = 0.466F1 + 0.198F2 + 0.107F3

Using the above formulas, the F values for each variety were calculated and ranked. The results indicate that LZ24 exhibited the best drought tolerance, followed by JZ12, while HH and JA60 showed the poorest drought tolerance accordingly (Table 5).

### 2.5. Comprehensive Evaluation and Cluster Analysis of Drought Tolerance

Through the calculation of the membership function values, the drought tolerance levels and comprehensive rankings of each variety were ascertained. The greater the value of the membership function, the stronger the drought tolerance capacity of the variety. LZ24 boasted the highest membership function value, which signified that it possessed the optimal drought tolerance ability. In contrast, JZ12 and HH had the lowest membership function values, suggesting that they had the poorest drought tolerance. Moreover, JA60 showed complete wilting under D3 level (Table 6).

**Figure 4:**
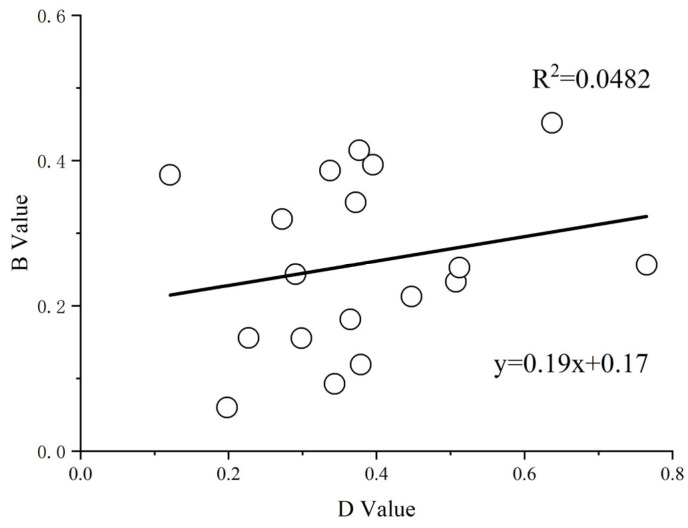
Regression analysis of (B) values versus (D) values.

**Figure 5:**
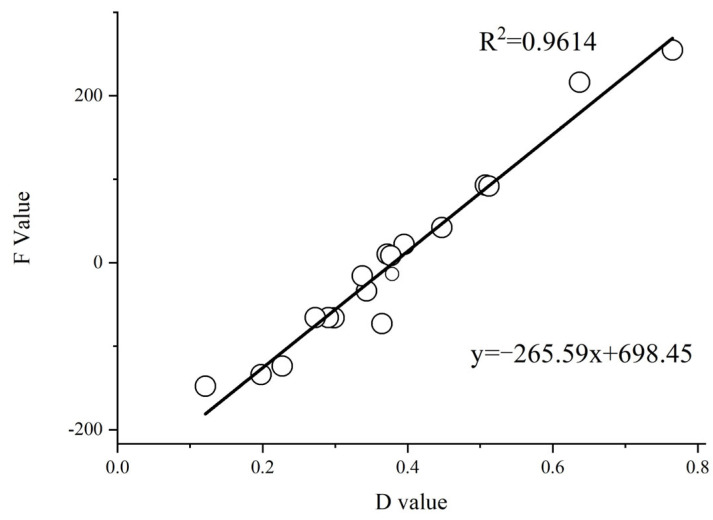
Regression analysis of (F) values versus (D) values.

Correlation analysis was conducted between the (D) values and (B) values, as well as between the (D) values and (F) values. A significant positive correlation between the (D) values and (F) values (R^2^ = 0.9614; *p* < 0.01) was observed, indicating that the two evaluation methods are feasible and consistent (Figure 4 and Figure 5). Subsequently, a systematic cluster analysis was performed using the (D) values and (F) values, categorizing the 19 sorghum varieties into three distinct groups:

Group I: Strong drought-tolerant varieties, including LZ24 and JZ12.

Group II: Moderately drought-tolerant varieties, including nine varieties: LZ23, LBH, JL6, KS, Hunni, HN, DXN, LZ21, and LZ27.

**Figure 6:**
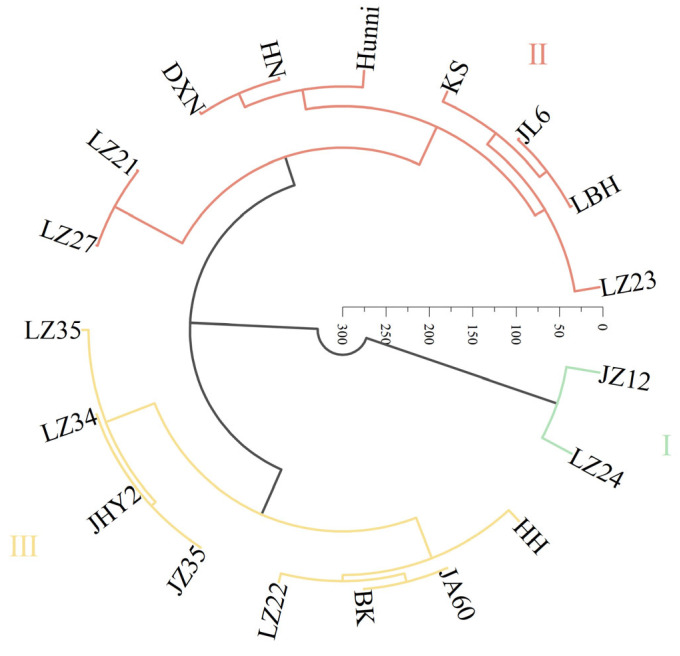
Systematic clustering based on drought tolerance of 19 sorghum varieties. Those with similar squared Euclidean distances were divided into one cluster. Different colors represent a cluster.

Group III: Drought-sensitive varieties. This group included eight varieties: LZ35, LZ24, JHY2, JZ35, LZ22, BK, JA60, and HH (Figure 6).

## 3. Discussion

Due to the lengthy growth cycle of crops, the establishment and application of rapid identification methods during germination and seedling stages can significantly enhance the efficiency of drought resistance research. Germination is a critical phase in the crop life cycle, regulated by various factors such as hormones, light, temperature, and moisture [23]. However, water availability is the most limiting factor for seed germination and crop growth in arid and semi-arid regions. During germination, drought stress adversely affects the metabolic processes of seed germination, thereby reducing germination rates and ultimately delaying seedling establishment [24]. Under drought stress, germination rate and germination potential were significantly reduced. Therefore, in the present study, 19 sorghum varieties were ranked, with DXN ranking first in germination potential and rate. However, since this variety exhibited low germination potential and germination rate under normal conditions (36.67% and 51.33%, respectively), its high relative germination potential and relative germination rate suggest that single germination-stage indicators cannot solely serve as reliable drought tolerance indicators. Therefore, further observation of growth indicators during the seedling stage is required. In regions where drought stress is prevalent, seedling mortality is a common issue, and the early stages of crop growth, germination, emergence, and seedling establishment are likely the most vulnerable to drought stress [25]. Therefore, conducting drought tolerance screening during the germination and seedling stages is of critical importance for sorghum crops.

### 3.1. Screening of Drought Resistance Indicators in Sorghum Under Drought Stress

Crop drought resistance is a complex quantitative trait regulated by both genetic factors and environmental conditions [26]. As the critical linkage between above-ground and underground plant parts, the root system serves as the primary organ perceiving drought stress in the soil environment. To combat drought conditions, roots activate a series of adaptive strategies, including root distribution adjustment [27], biomass reallocation [28], and morphological modifications [29], which directly determine a plant’s drought tolerance or avoidance capacity. Research has demonstrated that root characteristics such as biomass, diameter, and length are key indicators for evaluating plant drought resistance [30], with total root length being confirmed as a particularly significant parameter in our study. Given that phenotypic traits directly reflect plant physiological activity [31], it is crucial to adopt systematic drought resistance indicators combined with robust evaluation methodologies for comprehensive plant assessment [32]. In this study, principal component analysis (PCA) was employed to reduce the dimensionality of multivariate data, consolidating numerous correlated traits into a smaller set of representative variables [33]. This approach has been successfully applied in drought resistance analysis and evaluation systems for crops like maize and wheat, effectively identifying key drought-resistant indicators [34,35,36]. By integrating above-ground and root morphological traits in sorghum, we selected 11 morphological parameters for drought resistance evaluation. Notably, these indicators exhibited significant intercorrelations (Figure 3), potentially leading to redundancy in stress resistance assessment. To address this, PCA was used to reduce the 11 indicators into three independent principal components. The analysis revealed that total root length and root surface area showed the highest loadings in the first principal component (F1), stem diameter contributed most significantly to the second component (F2), and leaf area exhibited prominent loading in the third component (F3). Consequently, total root length, stem diameter, and leaf area can be established as core evaluation criteria for screening drought-resistant sorghum at the seedling stage.

### 3.2. Methods for Screening Drought Tolerance in Sorghum

Drought resistance in crops is a complex process that cannot be assessed using a single analytical approach. The membership function method is employed to evaluate drought resistance based on comprehensive crop indices [37]. This method emphasizes the drought resistance of sorghum and provides significant importance in screening drought-tolerant varieties. The combination of multiple drought tolerance indicators, represented by the (D) value, can serve as an evaluation parameter to effectively and accurately identify the drought tolerance of millet [38,39] because a higher (D) value indicates stronger drought tolerance. Previous studies on crop drought tolerance have predominantly relied on single evaluation methods; however, the present study utilizes a comprehensive assessment framework that integrates the drought tolerance coefficient, membership function value, and principal component score to accurately rank drought tolerance in sorghum genotypes. The combination of these three analytical methods improves the accuracy and reliability of drought tolerance screening in sorghum under drought stress conditions. Notably, this study revealed a divergence between B values and D values in their assessment outcomes, whereas F values demonstrated high consistency with D values. The synergistic relationship between D values and F values in multi-index evaluation establishes a “standardization–dimensionality reduction-comprehensive evaluation” framework. While the former (D values) resolves data comparability issues, the latter (F values) extracts core features, and their integration significantly enhances the objectivity and accuracy of the evaluation. Consequently, we employed combined F values and D values for cluster analysis to classify sorghum varieties into distinct drought tolerance categories. This dual-parameter approach proved particularly effective in discriminating varying grades of drought resistance among cultivars.

## 4. Materials and Methods

### 4.1. Sorghum Varieties

This study consisted of 19 sorghum varieties widely cultivated in China, the United States, and Australia (Table 7)

### 4.2. Experimental Methods

A controlled study was conducted in 2023 at the Joint International Research Laboratory of Agriculture and Agri-Product Safety of the Ministry of Education at Yangzhou University. Four PEG concentration gradients were used: 0%, 15%, 20%, and 25% PEG-6000. (Corresponding to water potentials of 0, −2.95, −7.35, and −10.27 MPa at 25 °C) [40]. The seeds were selected based on the standard seed analysis procedures issued by the Ministry of Agriculture of the People’s Republic of China. For each variety, 100 sorghum seeds of uniform size were selected. The seeds were sterilized by soaking in 10% sodium hypochlorite for 10 min and then rinsed with distilled water until no pungent odor remained. The seeds were evenly placed in sterile petri dishes (150 mm in diameter) lined with double layers of filter paper. During germination, PEG solutions were applied to simulate drought stress conditions. To maintain a relatively stable level in the petri dishes, the evaporated PEG solution was replenished daily. After 7 days, the seedlings in the petri dishes were transferred to specially designed cultivation tanks (rectangular tanks with dimensions of 58.0 cm in length, 11.0 cm in width, and 9.0 cm in height, featuring perforated lids for easy planting). The tanks were filled with PEG solutions for stress treatment. The Hoagland nutrient solution (1:2000 dilution) was added to ensure adequate nutrient supply for seedling growth. The PEG solutions were prepared with the Hoagland nutrient solution to maintain the desired PEG concentration. The plant tanks were placed in a controlled climate chamber set at 25 °C, with a 12/12 h light/dark cycle and 60% relative humidity. The stress solution was replaced every 3 days in each petri dish. The seedlings were placed in the tanks for 20 days, and samples were collected on the 27th day after seed germination for relevant measurements. In the whole process of this study, normal hydroponic conditions (CK) served as the control. There were three replicates for each treatment in this study.

### 4.3. Measurements

#### 4.3.1. Sampling and Measurement

On the 4th and 7th days of germination, the germination potential and germination rate were measured, respectively. On the 27th day after germination, five seedlings from each treatment were sampled to measure root morphological parameters, above-ground fresh weight, root fresh weight, above-ground dry weight, root dry weight, relative chlorophyll content, plant height, stem diameter, and leaf area.

Root morphological parameters

The roots were thoroughly rinsed and scanned using a root scanner machine (Model SC-GX, Zhejiang Shangsheng Instrument, China Zhejiang Province). Subsequently, root analysis software (Zhejiang Shangsheng Instrument) was employed to analyze the total root length (RL), total root surface area (RSA), and total root volume (RV).

Fresh weight (FW)

The above-ground fresh weight (AFW) and root fresh weight (RFW) of sample plants were measured separately using a precision balance (Model PTX-FA110F, Huazhi, China Fujian Province).

Dry weight (DW)

The samples were baked at 105 °C for 30 min and dried at 80 °C for 7 days, and then the above-ground dry weight (ADW) and below-ground dry weight (RDW) were measured.

Relative chlorophyll content (SPAD)

The SPAD value was measured using a portable chlorophyll meter (SPAD-502, Konica Minolta Japanese, Saitama Prefecture) on the 27th day after germination sampling.

Stem diameter (SD)

The stem diameter at the basal internode of sorghum was measured using a vernier caliper in millimeters (mm).

Plant height (PH)

The plant height was measured from the base of the sorghum plant to the uppermost expanded leaf using a measuring tape in centimeters (cm).

#### 4.3.2. Calculation Methods for Indicators

Germination potential (GP)

Germination Potential = Number of seeds germinated on the 4th day/Total number of tested seeds × 100%

Germination rate (GR) [41]

Germination Rate = Number of seeds germinated on the 7th Day/Total Number of Tested Seeds × 100%

Relative germination potential (RGP)

Relative germination potential (%) = Germination potential of the treatment group/Germination potential of the control group (CK).

Relative germination rate (RGR) [42]

Relative germination rate (%) = Germination rate of the treatment group/Germination rate of the control group (CK).

Leaf area (LA)

Leaf area = Leaf length × Leaf width × 0.75

### 4.4. Data Statistical and Analytical Methods

Data collection and calculation of averages were performed using Excel 2019. Statistical analyses, including difference analysis, correlation analysis, regression analysis, PCA, and cluster analysis, were performed using SPSS 25.0. Graphs were generated using Origin 2021.

Single drought resistance coefficient (a) [43]:

a = Value under drought stress/Value under normal conditions

Comprehensive drought resistance coefficient (B):$B=\sum_{i=1}^{n}\;a;$

Membership function value [μ(Xi)]: $\mu (X_{i})=\frac{X_{i}-X_{min}}{X_{max}-X_{min}};$ Xi: Average value of the i indicator. Xmax: Maximum average value across all varieties. *X*min: Minimum average value across all varieties. *i* = 1, 2, 3, …, n. *i* = 1, 2, 3…,n.

$W_{i}=\frac{{\mathrm{CV}}_{i}}{\sum_{i}^{n}{\mathrm{CV}}_{i}}$; CVi: The coefficient of variation of μ(*Xi*): Each tested material.

Drought tolerance measure (D) [44]:

$$D=\sum_{i=1}^{n}\;[\mu (X_{i}\times W_{i})]$$

Principal component analyses (PCA)

Principal components were extracted based on eigenvalues greater than 1 and cumulative contribution rates exceeding 85%. The principal component scores (F) were calculated.

Cluster analysis

Based on the principal component scores (F) and Drought tolerance measure (D), systematic cluster analysis was performed to classify the drought tolerance levels of the varieties.

## 5. Conclusions

The present study demonstrated that the 19 sorghum varieties exhibit varying levels of drought tolerance during both the germination and seedling stages under drought stress. Drought-related traits such as germination potential, germination rate, plant height, stem diameter, leaf area, and biomass were all negatively affected by drought stress, with germination potential being more severely impacted than germination rate. The PCA analysis revealed that root length, stem diameter, and leaf area can serve as reliable indicators for evaluating drought tolerance during seedling stage. The combined use of (F) and (D) values, along with cluster analysis, classified the 19 sorghum varieties into three drought tolerance levels: strong drought-tolerant, moderately drought-tolerant, and drought-sensitive. Based on the drought tolerance ranking and cluster analysis, the LZ24 and JZ12 varieties were identified as the most drought-tolerant varieties during seedling stage among the 19 sorghum genotypes evaluated. These varieties can be used for subsequent drought tolerance gene discovery and planting in arid areas.

## Figures and Tables

**Table 1:** Drought-tolerance-related indicators and rankings of sorghum varieties during germination under different PEG concentration treatments.

Cultivars	Germination Potential (%)				Germination Rate (%)				Relative Germination Potential			Relative Germination Rate			Ranking
	CK	D1	D2	D3	CK	D1	D2	D3	D1	D2	D3	D1	D2	D3	
LZ23	55.67 a	31.00 b	27.67 b	16.33 c	70.00 a	58.00 b	43.33 bc	38.33 c	0.56 a	0.50 a	0.29 b	0.83 a	0.62 ab	0.55 b	13
LZ24	87.00 a	87.00 a	74.33 b	25.67 c	91.33 a	92.33 a	81.00 a	42.33 b	1.00 a	0.85 b	0.30 c	1.01 a	0.89 a	0.46 b	3
LZ35	83.33 a	33.33 b	20.33 c	13.00 d	83.33 a	38.00 a	34.00 a	24.67 b	0.40 a	0.24 b	0.16 c	0.46 a	0.41 a	0.30 b	18
LZ34	74.00 a	36.00 b	32.67 bc	20.00 c	83.33 a	38.33 a	26.67 ab	26.33 b	0.49 a	0.44 a	0.27 b	0.46 a	0.32 a	0.32 a	17
LZ21	57.33 a	26.00 b	15.67 b	14.00 b	65.33 a	39.67 a	31.33 ab	21.33 c	0.45 a	0.27 b	0.24 b	0.61 a	0.48 a	0.33 b	16
LZ22	84.00 a	46.67 b	26.00 c	15.67 c	92.67 a	55.67 ab	48.00 ab	24.67 b	0.56 a	0.31 b	0.19 b	0.60 a	0.52 a	0.27 b	15
LZ27	50.67 a	17.67 b	14.00 b	13.00 b	64.67 a	27.33 a	23.00 b	14.00 b	0.35 a	0.28 a	0.26 a	0.42 a	0.36 ab	0.22 b	19
Hunni	92.33 a	71.33 b	70.33 c	45.67 c	96.00 a	78.33 ab	77.67 b	48.33 c	0.77 a	0.76 a	0.49 b	0.82 a	0.81 a	0.50 b	4
Bk	80.00 a	77.33 b	39.00 c	34.33 c	89.67 a	83.33 a	50.33 a	41.00 b	0.97 a	0.49 b	0.43 b	0.93 a	0.60 b	0.49 b	8
HN	86.00 a	81.33 b	52.33 c	31.33 c	89.67 a	91.67 a	72.00 a	35.00 b	0.95 a	0.61 b	0.36 c	0.98 a	0.80 b	0.39 c	6
LBH	61.67 a	38.67 b	34.33 c	34.00 c	84.00 a	56.33 ab	50.67 b	37.00 c	0.63 a	0.56 a	0.55 a	0.67 a	0.60 ab	0.44 b	12
KS	38.67 a	26.00 ab	25.67 bc	16.67 c	43.00 a	32.33 a	30.33 b	17.67 b	0.67 a	0.66 a	0.43 b	0.75 a	0.71 a	0.41 b	10
JZ12	76.00 a	47.33 b	43.00 bc	35.33 c	85.33 a	84.00 a	80.67 a	44.33 b	0.62 a	0.57 ab	0.46 b	0.98 a	0.96 a	0.53 b	5
JZ35	45.67 a	32.67 a	31.33 a	28.33 a	43.67 a	43.33 a	43.33 a	32.33 b	0.72 a	0.69 a	0.62 a	0.99 a	0.99 a	0.75 a	2
JA60	60.33 a	32.00 b	28.67 bc	24.33 c	75.00 a	38.67 ab	32.33 b	31.67 c	0.53 a	0.48 a	0.40 a	0.52 a	0.43 a	0.42 a	14
JHY2	60.00 a	40.33 b	34.33 b	29.33 b	76.33 a	49.00 ab	48.00 b	43.67 c	0.67 a	0.57 a	0.49 a	0.64 a	0.63 a	0.57 a	11
HH	63.67 a	44.00 b	38.67 bc	38.00 c	71.33 a	59.33 ab	53.67 b	40.00 c	0.69 a	0.61 a	0.60 a	0.83 a	0.75 a	0.56 b	7
DXN	36.67 a	33.33 ab	32.67 b	30.33 b	51.33 a	48.33 a	43.00 b	39.00 b	0.91 a	0.89 a	0.83 a	0.94 a	0.84 a	0.81 a	1
JL6	43.33 a	42.33 a	27.00 a	22.33 a	71.33 a	48.33 a	42.67 a	32.33 b	0.98 a	0.64 b	0.53 b	0.68 a	0.60 a	0.45 b	9

Different lowercase letters in the same column indicate statistical difference at the 0.05 probability level. CK, D1, D2, and D3 in the table represent 0%, 15%, 20%, and 25% PEG-6000 solutions, respectively. Different lowercase letters indicate significant differences between different drought level treatments of the same sorghum variety at a *p* < 0.05 level.

**Table 2:** Coefficients of variation of the measured values of each attribute in sorghum varieties treated with different PEG concentrations.

Parameters	PEG Treatment	Mean	Standard Deviation	CV (%)
PH (cm)	CK	28.09	10.51	0.37
	D1	20.02	4.11	0.21
	D2	12.48	3.60	0.29
	D3	5.96	2.47	0.41
SD (mm)	CK	2.26	0.78	0.35
	D1	1.69	0.42	0.25
	D2	1.42	0.30	0.21
	D3	1.16	0.27	0.24
LA (cm^2^)	CK	22.80	20.44	0.90
	D1	10.28	4.30	0.42
	D2	5.99	3.67	0.61
	D3	1.99	2.38	1.20
AFW (mg)	CK	542.84	592.97	1.09
	D1	202.43	120.25	0.59
	D2	116.45	80.30	0.69
	D3	33.95	19.91	0.59
ADW (mg)	CK	44.91	30.10	0.67
	D1	30.26	24.65	0.81
	D2	20.44	15.09	0.74
	D3	7.38	3.24	0.44
RFW (mg)	CK	322.00	319.95	0.99
	D1	139.19	66.42	0.48
	D2	106.96	54.16	0.51
	D3	57.05	12.76	0.22
RDW (mg)	CK	62.55	66.44	1.06
	D1	19.73	8.11	0.41
	D2	14.90	4.94	0.33
	D3	9.85	4.36	0.44
SPAD	CK	28.26	5.48	0.19
	D1	28.27	2.67	0.09
	D2	21.51	4.17	0.19
	D3	14.21	5.56	0.39
RL (cm)	CK	204.15	205.84	1.01
	D1	69.45	37.32	0.54
	D2	41.07	23.17	0.56
	D3	14.13	7.69	0.54
RSA (cm^2^)	CK	21.22	24.84	1.17
	D1	8.15	4.92	0.60
	D2	4.12	2.53	0.61
	D3	1.49	0.70	0.47
RV (mm^3^)	CK	163.12	227.54	1.39
	D1	99.07	80.72	0.81
	D2	33.25	20.93	0.63
	D3	14.65	9.03	0.62

CK, D1, D2, and D3 in the table represent 0%, 15%, 20%, and 25% PEG-6000 solutions, respectively. PH, plant height; SD, stem diameter; LA, leaf area; AFW, above-ground fresh weight; ADW, above-ground dry weight; RFW, root fresh weight; RDW, below-ground dry weight; SPAD, relative chlorophyll content; RSA, total root surface area; RL, total root length; RV, total root volume.

**Table 3:** Ranking of single drought tolerance coefficients and combined drought tolerance coefficients for each index at the seedling stage of sorghum.

Cultivars	Single Drought Resistance Coefficient (a)											Comprehensive Drought Resistance Coefficient (B)	Ranking
	PH (cm)	SD (mm)	LA (cm^2^)	AFW (mg)	ADW (mg)	RFW (mg)	RDW (mg)	SPAD	RL (cm)	RSA (cm^2^)	RV (mm^3^)		
LZ23	0.31	0.58	0.14	0.48	0.13	0.17	0.14	0.35	0.09	0.09	0.08	0.21	12
LZ24	0.43	0.51	0.17	0.57	0.17	0.17	0.22	0.30	0.19	0.17	0.18	0.26	8
LZ35	0.12	0.43	0.55	0.42	0.04	0.07	0.16	0.18	0.05	0.06	0.09	0.18	13
LZ34	0.17	0.76	0.07	0.25	0.05	0.09	0.11	0.21	0.03	0.07	0.06	0.16	15
LZ21	0.22	0.83	0.06	0.75	0.10	0.13	0.19	0.28	0.08	0.09	0.08	0.23	11
LZ22	0.09	0.57	0.20	0.39	0.03	0.03	0.13	0.28	0.04	0.04	0.07	0.16	14
LZ27	0.26	0.66	0.13	0.81	0.12	0.21	0.17	0.44	0.06	0.08	0.09	0.25	9
Hunni	0.14	0.38	0.02	0.27	0.02	0.12	0.06	0.07	0.02	0.01	0.01	0.09	17
Bk	0.07	0.27	0.003	0.18	0.01	0.09	0.04	0.04	0.01	0.01	0.00	0.06	18
HN	0.14	0.39	0.01	0.39	0.02	0.25	0.11	0.06	0.02	0.01	0.02	0.12	16
LBH	0.20	0.81	0.09	0.59	0.13	0.58	0.58	0.49	0.12	0.11	0.42	0.34	6
KS	0.25	0.72	0.17	0.64	0.24	0.92	0.45	0.35	0.19	0.28	0.51	0.39	3
JZ12	0.50	0.71	0.34	0.72	0.32	0.55	0.59	0.30	0.30	0.24	0.85	0.45	1
JZ35	0.18	0.67	0.12	0.47	0.11	0.23	0.57	0.09	0.08	0.17	0.23	0.24	10
JA60													19
JHY2	0.23	0.89	0.04	0.27	0.09	0.38	0.48	0.42	0.27	0.33	0.43	0.32	7
HH	0.15	0.51	0.04	0.81	0.08	0.43	0.48	0.22	0.43	0.61	0.81	0.38	5
DXN	0.35	0.66	0.19	0.48	0.21	0.13	0.71	0.23	0.55	0.60	0.53	0.39	4
JL6	0.29	0.62	0.16	0.57	0.23	0.71	0.52	0.20	0.61	0.58	0.47	0.41	2

PH, plant height; SD, stem diameter; LA, leaf area; AFW, above-ground fresh weight; ADW, above-ground dry weight; RFW, root fresh weight; RDW, below-ground dry weight; SPAD, relative chlorophyll content; RSA, total root surface area; RL, total root length; RV, total root volume.

**Table 4:** Eigenvectors and contributions of principal components of drought tolerance indexes of sorghum varieties.

Attribute	Principal Component		
	F1	F2	F3
PH (cm)	0.88	0.10	0.06
SD (mm)	0.25	0.84	−0.26
LA (cm^2^)	0.06	−0.04	0.85
AFW (mg)	0.69	−0.15	−0.09
ADW (mg)	0.90	0.21	0.15
RFW (mg)	0.63	0.52	−0.21
RDW (mg)	0.52	−0.33	−0.45
SPAD	0.35	0.68	0.31
RL (cm)	0.97	−0.02	0.07
RSA (cm^2^)	0.91	−0.33	0.07
RV (mm^3^)	0.66	−0.68	0.03
Characteristic value	5.13	2.18	1.18
Contribution rate%	46.59	19.84	10.74
Cumulative contribution rate%	46.59	66.43	77.18
Factor weight	0.60	0.26	0.14

PH, plant height; SD, stem diameter; LA, leaf area; AFW, above-ground fresh weight; ADW, above-ground dry weight; RFW, root fresh weight; RDW, below-ground dry weight; SPAD, relative chlorophyll content; RSA, total root surface area; RL, total root length; RV, total root volume.

**Table 5:** Score values and ranking of principal components of drought tolerance in sorghum varieties.

Cultivars	F Value	Ranking
LZ23	42.28	5
LZ24	254.61	1
LZ35	−72.83	15
LZ34	−66.01	14
LZ21	93.16	3
LZ22	−123.81	16
LZ27	91.95	4
Hunni	−33.84	11
BK	−133.95	17
HN	−13.60	9
LBH	10.29	7
KS	21.95	6
JZ12	216.13	2
JZ35	−65.57	12
JA60		19
JHY2	−65.82	13
HH	−147.79	18
DXN	−15.70	10
JL6	8.55	8

**Table 6:** Drought tolerance affiliation function analysis of sorghum varieties.

Cultivars	Membership Function Value (μ)					Drought Tolerance Measure (D)	
	μ1	μ2	μ3	μ4	μ5	Value	Ranking
LZ23	0.38	0.80	0.52	0.17	0.10	0.45	5
LZ24	0.95	0.74	0.52	0.70	0.00	0.77	1
LZ35	0.13	0.47	1.00	1.00	0.65	0.36	10
LZ34	0.14	0.70	0.46	0.22	0.20	0.30	13
LZ21	0.49	1.00	0.26	0.09	1.00	0.51	4
LZ22	0.00	0.53	0.82	0.18	0.64	0.23	16
LZ27	0.51	0.83	0.46	0.18	0.65	0.51	3
Hunni	0.24	0.70	0.28	0.38	0.18	0.34	11
BK	0.01	0.62	0.30	0.28	0.09	0.20	17
HN	0.32	0.68	0.12	0.63	0.27	0.38	7
LBH	0.47	0.39	0.15	0.37	0.95	0.37	9
KS	0.48	0.38	0.34	0.34	0.49	0.40	6
JZ12	1.00	0.16	0.68	0.10	0.42	0.64	2
JZ35	0.33	0.25	0.00	0.85	0.57	0.29	14
JA60							19
JHY2	0.26	0.41	0.19	0.33	0.20	0.27	15
HH	0.15	0.00	0.38	0.00	0.37	0.12	18
DXN	0.34	0.46	0.48	0.07	0.25	0.34	12
JL6	0.44	0.37	0.41	0.29	0.17	0.38	8

**Table 7:** Sorghum varieties used in this study.

Cultivars	Breeding Country	Abbreviation	Source
Longza23	China	LZ23	Heilongjiang Academy of Agricultural Sciences
Longza24	China	LZ24	Heilongjiang Academy of Agricultural Sciences
Longza35	China	LZ35	Heilongjiang Academy of Agricultural Sciences
Longza34	China	LZ34	Heilongjiang Academy of Agricultural Sciences
Longza21	China	LZ21	Heilongjiang Academy of Agricultural Sciences
Longza22	China	LZ22	Heilongjiang Academy of Agricultural Sciences
Longza27	China	LZ27	Heilongjiang Academy of Agricultural Sciences
Hunnigreen	USA	Hunni	Fuyuanlai Limited
Big Kahuna	USA	BK	Barenbrug
Hainiu	Australian	HN	Fuyuanlai Limited
Lvbaohong	China	LBH	Shouhe Agriculture
Kangsi	China	KS	Shouhe Agriculture
Jingza12	China	JZ12	Shouhe Agriculture
Jingza35	China	JZ35	Shouhe Agriculture
Jiaai60	China	JA60	Shouhe Agriculture
Jinghongying2	China	JHY2	Shouhe Agriculture
Huanghe	China	HH	Shouhe Agriculture
Dingxinnuo	China	DXN	Shouhe Agriculture
Jiliang6	China	JL6	Shouhe Agriculture

## Data Availability

The original contributions presented in this study are included in the article. Further inquiries can be directed to the corresponding authors.
